# Comparison of conbercept and ranibizumab for the treatment of idiopathic choroidal neovascularization using optical coherence tomography angiography and multifocal electroretinography

**DOI:** 10.3389/fphar.2026.1854440

**Published:** 2026-06-24

**Authors:** Gaixia Zhai, Lin Zhu, Yuanzhen Su, Zuofen Wang

**Affiliations:** Zibo Central Hospital, Zibo, China

**Keywords:** conbercept, efficacy, idiopathic choroidal neovascularization, multifocal electroretinography, ranibizumab, vascular endothelial growth factor

## Abstract

**Objective:**

This study aimed to compare the efficacies of conbercept and ranibizumab for the treatment of idiopathic choroidal neovascularization (ICNV) using optical coherence tomography angiography (OCTA) and multifocal electroretinography (mfERG).

**Methods:**

Sixty patients with ICNV were retrospectively assessed and followed up in this retrospective comparative cohort study. Thirty patients were assigned to the conbercept group, and 30 patients were assigned to the ranibizumab group. Patients in both groups were treated with 1+ pro re nata. Best-corrected visual acuity (BCVA), macular central retinal thickness (CRT), and CNV blood flow area before treatment and at 1, 3, 6, and 12 months after treatment were obtained. The first positive peak (P1) amplitude density in ring 1 and intraocular pressure (IOP) were recorded at baseline and the last follow-up.

**Results:**

The follow-up duration was 12 months. BCVA (logMAR) improved at 1, 3, 6, and 12 months after the injection, and the CRT and CNV blood flow area significantly decreased in both groups relative to their values before treatment (p < 0.05). The P1 amplitude densities in ring 1 at baseline were 50.97 ± 8.05 nv/deg^2^ and 52.50 ± 9.71 nv/deg^2^ in the conbercept and ranibizumab groups, respectively (p = 0.5080). The change in the P1 amplitude density from baseline to the last follow-up visit was statistically significant (p < 0.05) in both groups. No significant changes were noted in the BCVA, IOP, CRT, CNV blood flow area, and P1 amplitude density after treatment from the baseline for the two groups. The number of injections was 2.00 ± 1.05 for the conbercept group and 2.67 ± 1.06 for the ranibizumab group (p = 0.0077). No serious ocular complications such as endophthalmitis and systemic adverse reactions such as myocardial infarction or cerebral infarction occurred in both groups.

**Conclusion:**

Compared with intravitreal ranibizumab, intravitreal conbercept had a similar therapeutic effect in improving the structure and function on ICNV but with fewer injections.

## 1 Introduction

Choroidal neovascularization (CNV) is an abnormal hyperplasia of a network of blood vessels from the choroidal vascular layer that extends beyond the Bruch’s membrane to the retinal pigment epithelium (RPE) and/or sensory layer (Lai et al., 2018; Chu et al., 2017). The main causes of CNV in patients younger than 50 years include pathologic myopia, central serous chorioretinopathy (CSC), trauma, cytomycosis, angioid streaks, and other hereditary ocular diseases (Cohen et al., 1996; Yin et al., 2016). The etiology of CNV is unknown in some patients aged < 50 years, and this type of CNV is called idiopathic choroidal neovascularization (ICNV) (Ho et al., 1995). ICNV accounts for 17% of the cases of patients with CNV younger than 50 years (Cohen et al., 1996). ICNV results in severely impaired vision because of repeated exudation and hemorrhage involving the fovea. ICNV is a major cause of visual acuity loss and visual dysfunction in young individuals (David and Lawrence, 2006; Bing et al., 2018).

The development of CNV is associated with an increase in the intraocular vascular endothelial growth factor (VEGF) level (Kumar et al., 2004). Similar to CNV secondary to age-related macular degeneration (AMD), anti-VEGF treatment is currently the main regimen for ICNV (Liu et al., 2016). By inhibiting VEGF, neovascularization can be effectively inhibited, and vascular leakage can be reduced. At present, the commonly used anti-VEGF drugs for ICNV include aflibercept, conbercept, and ranibizumab. Ranibizumab is a humanized monoclonal immunoglobulin G1 antibody that binds to all VEGF-A subtypes. As a recombinant fusion protein, conbercept contains extracellular domain 2 of VEGF receptor (VEGFR)-1 and extracellular domains 3 and 4 of VEGFR-2. In addition to VEGF-A, it can bind to VEGF-B, VEGF-C, and placental growth factor (PIGF). Some studies have reported that conbercept has a stronger binding affinity for VEGF-A than ranibizumab (Lu and Sun, 2015; Zhang et al., 2011). A previous study found similar therapeutic effects of intravitreal ranibizumab and bevacizumab for treating ICNV (Wu Q. et al., 2020). A comparative study of conbercept and ranibizumab for the treatment of macular edema (ME) secondary to branch retinal vein occlusion reported similar efficacies (Li et al., 2017). In most studies, primary measures including best-corrected visual acuity (BCVA) and macular central retinal thickness (CRT) are used to evaluate the clinical efficacy of anti-VEGF agents for treating ICNV, however, they do not reflect the function of the retina.

As a noninvasive imaging technique, for example, optical coherence tomography angiography (OCTA) can estimate retinal blood flow through the superficial capillary plexus (SCP) and deep capillary plexus (DCP), among others (Jia et al., 2014). It is useful for observing morphological changes in blood vessels and neovascularization and blood perfusion in different layers of the retina. Multifocal electroretinography (mfERG) is the most effective electrophysiological method for measuring posterior polar retinal function. Its main advantage is the evaluation of visual function in patients with macular disease, and it is effective for detecting early micromacular disease that lacks morphological changes. MfERG can be used to quantitatively evaluate the efficacy of anti-VEGF therapy for the treatment of ICNV. Few studies have considered OCTA and mfERG when evaluating the efficacy of anti-VEGF therapy for treating ICNV. OCTA combined with mfERG can be used to observe retinal morphology on the one hand and quantitatively observe retinal function on the other hand. Several studies have utilized mfERG to evaluate retinal functional changes after intravitreal therapy for diabetic macular edema, including anti-VEGF (Zübeyir et al., 2021) and dexamethasone implant (Paris et al., 2024).

Studies comparing the efficacies of intravitreal conbercept and ranibizumab for the treatment of ICNV using OCTA and mfERG are lacking. This retrospective comparative cohort study investigated and followed 60 patients with ICNV who received intravitreal injections of conbercept or ranibizumab at our hospital. We aimed to compare the efficacies of intravitreal injections of conbercept and ranibizumab for the treatment of ICNV using OCTA and mfERG.

## 2 Materials and methods

### 2.1 Study protocol

The Medical Ethics Committee of Zibo Central Hospital (202001006) approved this study, which followed the principles of the Declaration of Helsinki. This was a retrospective comparative cohort study. The risks and benefits of intravitreal injections were explained to all patients before treatment initiation. Each patient provided informed consent before surgery.

### 2.2 Patients

The clinical data of 60 patients (60 eyes) with ICNV admitted to the Department of Ophthalmology of Zibo Central Hospital between January 2020 and October 2022 were retrospectively analyzed. The patients were divided into two groups: the conbercept and ranibizumab groups. Patients in both groups were treated with 1+ pro re nata (PRN). Although a 3-month loading dose is standard for ranibizumab in some indications, a 1+PRN regimen was chosen in this study for both groups because ICNV typically shows a favorable response to anti-VEGF therapy, and our clinical experience suggested that a single initial injection followed by PRN retreatment could achieve comparable anatomical and functional outcomes while reducing the frequency of injections and follow-up visits.

The inclusion criteria were having newly diagnosed ICNV, having undergone comprehensive ophthalmic examination, and age of <50 years. The exclusion criteria were as follows: previous eye surgery or vitreous injection; refractive error of ≥6.00D or axial length of ≥26.0 mm; and/or history of retinal vein obstruction, glaucoma, retinal detachment, or diabetic retinopathy. The characteristics of each study group before treatment are shown in Table 1.

**TABLE 1:** Baseline characteristics of the patients with idiopathic choroidal neovascularization.

Characteristics	Conbercept group (N = 30)	Ranibizumab group (N = 30)	P
Age (years)	32.33 ± 7.17	33.23 ± 7.51	0.6366
Gender, n (Male/Female)	14/16	15/15	0.7961
Eye type, n (Left/Right)	13/17	16/14	0.4383
Disease duration (from symptom onset to first injection) (d)	8.90 ± 3.43	8.37 ± 3.21	0.5364
IOP, mmHg (mean ± SD)	14.90 ± 2.62	13.87 ± 2.93	0.1553
BCVA (log MAR)	0.44 ± 0.13	0.42 ± 0.13	0.6381
CRT (µm)	305.9 ± 37.54	309.1 ± 37.32	0.7418
CNV flow area (mm2)	0.22 ± 0.06	0.21 ± 0.06	0.5340
P1 amplitude density (nv/deg2)	50.97 ± 8.05	52.50 ± 9.71	0.5080

Abbreviations: BCVA, best-corrected visual acuity; IOP, intraocular pressure; CRT, central retinal thickness; CNV, choroidal neovascularization.

### 2.3 Examination and treatment

All patients completed relevant examinations, including intraocular pressure (IOP), BCVA (logMAR), slit lamp biomicroscopy, ocular fundus examination, and OCTA (Optovue Inc, Fremont, CA, USA) before and after treatment. All patients underwent mfERG at the first visit and last follow-up. CRT and CNV blood flow area were measured using OCTA. IOP was measured using noncontact tonometry. Fluorescence fundus angiography (FFA) (Spectralis, Heidelberg, Germany) was feasible if necessary.

All patients received intravitreal injections of conbercept (0.05 mL/0.5 mg; Chengdu Kanghong Biotechnology Co., Ltd.) or ranibizumab (0.05 mL/0.5 mg; Lucentis; Genentech, Inc., South San Francisco, CA, USA) and were followed up monthly for at least 12 months. The treatment was chosen by the attending physician after discussion with the patient, taking into account the patient’s financial situation and drug availability, rather than based on disease severity or baseline characteristics.

The RETIscan multifocal ERG Version 3.15 system (Roland Company, Germany) was used for mfERG. All patients were examined only after pupil dilation using tropicamide (0.5%, Santen, Japan). The recording electrode was a corneal contact lens electrode. For analysis, mfERG responses were grouped into five concentric rings around the fovea (rings 1–5), with ring 1 representing the foveal response. Intravitreal injections were administered in a sterile operating room depending on the intraocular surgery. After injection, all patients were asked to administer tobramycin dexamethasone eye drops four times a day for 7 days. The criteria for reinjection included an increase in CRT by > 50 μm, an increase in the CNV blood flow area, and a decrease in BCVA by more than one row and new CNV lesions.

### 2.4 Observation parameters

The changes in BCVA, IOP, CRT, CNV blood flow area, and the first positive peak (P1) amplitude density in ring 1 after treatment from the baseline and the differences between the study groups were determined.

### 2.5 Statistical analysis

Quantitative data are expressed as mean ± standard deviation (SD). All continuous variables were tested using the Anderson–Darling test. The results showed that CRT, P1 amplitude density in ring 1, and IOP were normally distributed, while BCVA (logMAR), CNV blood flow area, and the number of injections were not normally distributed. BCVA, CRT, and CNV blood flow area were repeated measurement data (baseline and at 1, 3, 6, 12 months). Within-group comparisons: For normally distributed data, one-way repeated measures ANOVA was used (Greenhouse–Geisser correction applied when sphericity was violated). For non-normally distributed data, the Friedman test with *post hoc* analysis was used. Between-group comparisons at each time point: The independent t-test was used for normally distributed data, and the Mann-Whitney U test for non-normally distributed data. All statistical analyses were performed using GraphPad Prism 9 statistical software. Age was normally distributed and compared using the independent t-test. Sex and eye type were compared using the chi-square test. The number of injections was not normally distributed, so the Mann–Whitney U test was used for comparison. P-values of <0.05 denoted statistical significance.

## 3 Results

### 3.1 Baseline characteristics

Of the 30 patients (30 eyes) in the conbercept group, 14 were male and 16 were female. The mean age of the patients was 32.33 ± 7.17 years. The mean BCVA was 0.44 ± 0.13 logMAR. The mean IOP was 14.90 ± 2.62 mmHg. The mean CRT was 305.9 ± 37.54 µm. The mean CNV flow area was 0.22 ± 0.06 mm^2^. The mean P1 amplitude density in ring 1 was 50.97 ± 8.05 nv/deg^2^.

In the ranibizumab group, the mean age of the patients was 33.23 ± 7.51 years. The mean BCVA was 0.42 ± 0.13 logMAR. The mean IOP was 13.87 ± 2.93 mmHg. The mean CRT was 309.1 ± 37.32 µm. The mean CNV flow area was 0.21 ± 0.06 mm^2^. The mean P1 amplitude density in ring 1 was 52.50 ± 9.71 nv/deg^2^. The baseline characteristics of the patients are shown in Table 1.

### 3.2 Best-corrected visual acuity

**FIGURE 1:**
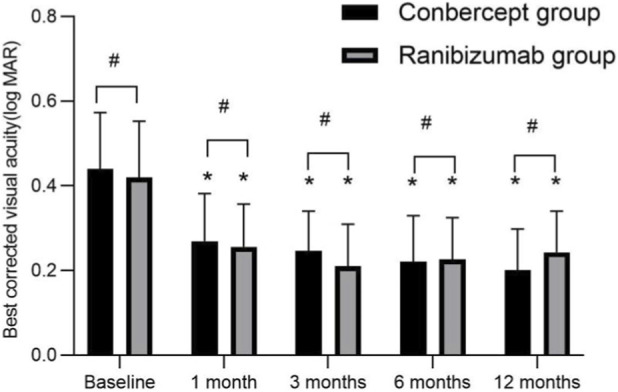
Best-corrected visual acuities of the conbercept and ranibizumab groups before and after treatment. * p < 0.05 compared with baseline. ^#^ p > 0.05 in the conbercept and ranibizumab groups. Data are expressed as mean ± SD.

In the conbercept group, the baseline BCVA improved from 0.44 ± 0.13 logMAR to 0.27 ± 0.11 logMAR, 0.25 ± 0.09 logMAR, 0.22 ± 0.11 logMAR, and 0.20 ± 0.10 logMAR at 1, 3, 6, and 12 months after injection (Table 2). For the ranibizumab group, the baseline BCVA improved from 0.42 ± 0.13 logMAR to 0.26 ± 0.10 logMAR, 0.21 ± 0.10 logMAR, 0.23 ± 0.10 logMAR, 0.24 ± 0.10 logMAR at 1, 3, 6, and 12 months after injection (Table 2). No significant differences were noted between the BCVAs of the two groups at 1, 3, 6, and 12 months after treatment (p = 0.6990, p = 0.1522, p = 0.6954, and p = 0.0574, respectively). The BCVAs of the two groups are shown in Figure 1.

**TABLE 2:** Outcomes in the conbercept and ranibizumab groups before and after treatment.

Indices (mean ± SD)	Time	Conbercept (N = 30)	Ranibizumab (N = 30)	P
BCVA (logMAR)	Preoperative	0.44 ± 0.13	0.42 ± 0.13	0.6381
	1 month	0.27 ± 0.11^*^	0.26 ± 0.10^*^	0.6990
	3 months	0.25 ± 0.09^*^	0.21 ± 0.10^*^	0.1522
	6 months	0.22 ± 0.11^*^	0.23 ± 0.10^*^	0.6954
	12 months	0.20 ± 0.10^*^	0.24 ± 0.10^*^	0.0574
CRT (μm)	Preoperative	305.9 ± 37.54	309.1 ± 37.32	0.7418
	1 month	267.3 ± 32.24^*^	265.2 ± 33.54^*^	0.6514
	3 months	248.4 ± 22.56^*^	244.8 ± 18.46^*^	0.4976
	6 months	241.9 ± 17.49^*^	237.1 ± 11.97^*^	0.2230
	12 months	244.9 ± 23.12^*^	241.4 ± 14.09^*^	0.8860
CNV area (mm^2^)	Preoperative	0.22 ± 0.06	0.21 ± 0.06	0.5340
	1 month	0.13 ± 0.03^*^	0.14 ± 0.05^*^	0.9971
	3 months	0.11 ± 0.03^*^	0.11 ± 0.03^*^	0.7758
	6 months	0.11 ± 0.04^*^	0.12 ± 0.04^*^	0.5018
	12 months	0.12 ± 0.04^*^	0.11 ± 0.03^*^	0.9557
P1 amplitude density (nv/deg^2^)	Preoperative	50.97 ± 8.05	50.97 ± 8.05	0.5080
	12 months	70.70 ± 9.20^*^	71.73 ± 7.65^*^	0.9557

Data are presented as mean ± SD., The far-right P column represents inter-group comparisons (conbercept vs. ranibizumab at each time point), while the asterisk (*) represents intra-group comparisons (post-operative vs. preoperative within the same group).

### 3.3 Central retinal thickness

**FIGURE 2:**
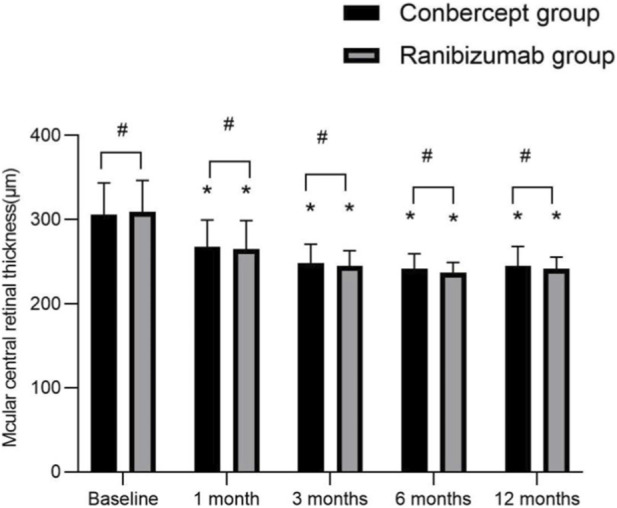
Macular central retinal thicknesses of the conbercept and ranibizumab groups before and after treatment. * p < 0.05 compared with baseline. ^#^ p > 0.05 in the conbercept versus ranibizumab groups. Data are expressed as mean ± SD.

CRT decreased significantly from 305.9 ± 37.54 µm to 267.3 ± 32.24 µm, 248.4 ± 22.56 µm, 241.9 ± 17.49 µm, and 244.9 ± 23.12 μm at 1, 3,6, and 12 months after injection, respectively, in the conbercept group (Table 2). In the ranibizumab group, CRT decreased significantly from 309.1 ± 37.32 µm to 265.2 ± 33.54 µm, 244.8 ± 18.46 µm, 237.1 ± 11.97 µm, and 241.4 ± 14.09 μm at 1, 3, 6, and 12 months after injection, respectively (Table 2). There were no significant differences between the CRTs of the two groups at 1, 3, 6, and 12 months after treatment (p = 0.6514, p = 0.4976, p = 0.2230, and p = 0.8860, respectively). The CRTs of the two groups are shown in Figure 2.

### 3.4 Choroidal neovascularization flow area

**FIGURE 3:**
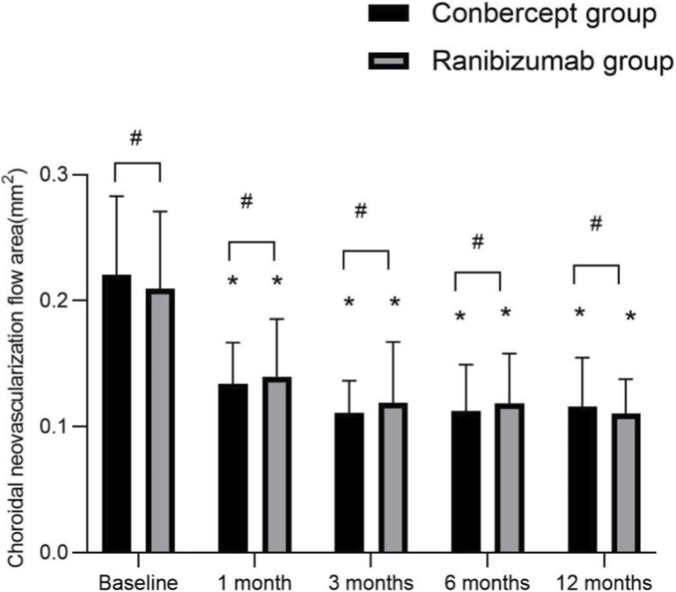
Choroidal neovascularization flow areas of the conbercept and ranibizumab groups before and after treatment. * p < 0.05 compared with baseline. ^#^ p > 0.05 in the conbercept and ranibizumab groups. Data are expressed as mean ± SD.

In the conbercept group, the baseline CNV blood flow area reduced significantly from 0.22 ± 0.06 mm^2^ to 0.13 ± 0.03 mm^2^, 0.11 ± 0.03 mm^2^, 0.11 ± 0.04 mm^2^, and 0.12 ± 0.04 mm^2^ at 1, 3, 6, and 12 months after the injection, respectively (Table 2). The baseline CNV blood flow area reduced significantly from 0.21 ± 0.06 mm^2^ to 0.14 ± 0.05 mm^2^, 0.12 ± 0.05 mm^2^, 0.12 ± 0.04 mm^2^, and 0.11 ± 0.03 mm^2^ at 1, 3, 6, and 12 months after the injection, respectively, in the ranibizumab group (Table 2). There were no significant differences between the CNV flow areas of the two groups at 1, 3, 6, and 12 months after the injection (p = 0.9971, p = 0.7758, p = 0.5018, and p = 0.9557, respectively). The CNV blood flow areas of the two groups are shown in Figure 3.

### 3.5 P1 amplitude density

**FIGURE 4:**
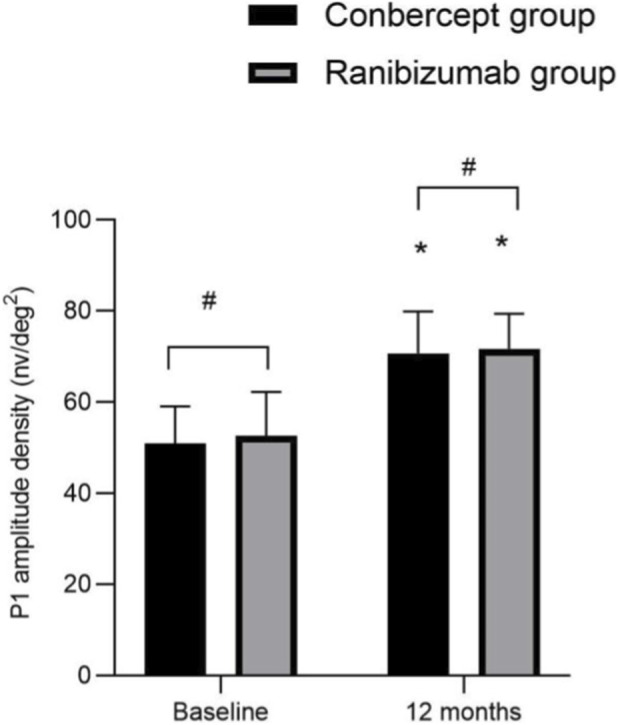
P1 amplitude densities of the conbercept and ranibizumab groups before and after treatment. * p < 0.05 compared with baseline. ^#^ p > 0.05 in the conbercept and ranibizumab groups. Data are expressed as mean ± SD.

The baseline P1 amplitude densities were 50.97 ± 8.05 nv/deg^2^ and 52.50 ± 9.71 nv/deg^2^ in the conbercept and ranibizumab groups, respectively (p = 0.5080) (Table 2). In both groups, a significant increase in the P1 amplitude density was observed at the last follow-up. In the conbercept and ranibizumab groups, the P1 amplitude densities were 70.70 ± 9.20 nv/deg^2^ and 71.73 ± 7.65 nv/deg^2^ at the last follow-up visit, respectively (p = 0.6381) (Table 2). The P1 amplitude densities of the two groups are shown in Figure 4.

### 3.6 Intraocular pressure

In the conbercept and ranibizumab groups, the mean IOPs were 14.47 ± 3.05 mmHg and 13.97 ± 2.81 mmHg at the last follow-up, respectively (p = 0.5115).

### 3.7 Number of injections

The number of injections was 2.00 ± 1.05 in the conbercept group and 2.67 ± 1.06 in the ranibizumab group (p = 0.0077).

### 3.8 Complications

No serious ocular complications such as endophthalmitis and systemic adverse reactions such as myocardial infarction and cerebral infarction occurred in both groups.

## 4 Discussion

Patients with CNV who are not treated promptly may soon experience irreversible vision loss. ICNV refers to CNV that is not accompanied by any other ocular diseases, such as pathological myopia, AMD, angioid streaks, toxoplasmosis, and histoplasmosis of the eye. ICNV is a major cause of visual acuity loss and visual dysfunction in young individuals. A previous study has shown that the serum VEGF levels of ICNV patients were higher than those of the control group (Yang et al., 2010). In the past, the main treatments for CNV were photodynamic therapy (PDT), transpupillary thermotherapy, and thermal laser photocoagulation; however, these did not effectively improve the vision of patients (Shi et al., 2014). VEGF is essential for the development of ICNV, and anti-VEGF drugs have demonstrated clinical efficacy for patients with ICNV. Some studies have reported that anti-VEGF therapy can significantly improve BCVA and reduce CRT in patients with ICNV (Sudhalkar et al., 2015; Inoue et al., 2010). In most of these studies on ICNV, anti-VEGF therapy (1+ PRN) has shown comparable efficacy (Zhang et al., 2012; Qi et al., 2010).

Optical coherence tomography angiography can identify the characteristics of CNV and has high specificity and sensitivity for the detection of neovascularization. All patients completed OCTA without indocyanine angiography or fluorescein angiography, thus avoiding the risks of angiography in the study. MfERG was invented by Sutter and Tran in 1992 (Li et al., 2022). As an ocular electrophysiologic test, electroretinography can help clinicians understand the health status of photoreceptors and bipolar cells in the retina (Hoffmann et al., 2021). Some studies have reported standard guidelines for the application of mfERG, which are still in clinical use today (Hoffmann et al., 2021). In recent years, mfERG has been widely used in clinical settings (Huang et al., 2021; Nagy et al., 2008; Lu et al., 2021). At present, only a few studies have used mfERG to assess the therapeutic effect of ICNV. In November 2016, ranibizumab was approved for the treatment of adults with CNV in the European Union (European Medicines Agency, 2016). A previous study found favorable effectiveness of an intravitreal injection of conbercept with a 1+ PRN regimen for the treatment of ICNV (Wu P. et al., 2020). Conbercept has a fourth VEGFR-2 binding domain, which improves the binding rate for VEGF and prolongs its half-life within the eye (Lu and Sun, 2015).

In this study, the baseline BCVA improved significantly in both groups after the injection. The baseline CRT and CNV blood flow areas reduced significantly at 1, 3, 6, and 12 months after treatment in the two groups. There were no significant differences in BCVA, IOP, CRT, and CNV flow area between the two groups before and after treatment. The number of injections was 2.00 ± 1.05 in the conbercept group and 2.67 ± 1.06 in the ranibizumab group (p = 0.0077). In both groups, a significant increase in the P1 amplitude density was observed at the last follow-up from the baseline. This suggests that intravitreal injections of conbercept or ranibizumab can help restore macular function. Conbercept has a stronger affinity for VEGF-A than ranibizumab and may be more useful for the treatment of ICNV. However, our study suggests that the efficacies of the two drugs are similar for the treatment of ICNV. Ranibizumab has a lower molecular weight than conbercept, which facilitates penetration of the site of action. In addition, the results of the study may be related to its small sample size and the retrospective nature. However, during a follow-up of at least 1 year, our results showed that the conbercept group received fewer injections than the ranibizumab group. The reason may be the long-term effect caused by the special structure (Kun et al., 2019). A study comparing the efficacy of conbercept with that of ranibizumab for the treatment of pathological myopic CNV reported that the treatment effects of the two drugs were similar (Chen et al., 2020). This is consistent with the results of this study. Intravitreal injections of anti-VEGF drugs may cause infection, bleeding, glaucoma, and cataracts. The risks are similar to those of intraocular surgery. One of the most common adverse effects of intravitreal drugs is increased IOP. In our study, no cases of increased IOP or systemic or ocular safety issues were observed. However, a 20-year-old boy showed low IOP and increased myopia after an intravitreal injection of ranibizumab. The IOP returned to normal, and the BCVA returned to preoperative levels 3 days after the addition of cycloplegic agents. No severe ophthalmologic complications occurred in either group. Furthermore, due to the retrospective design of this study, intraocular pressure (IOP) was measured only at baseline and at the last follow-up visit (12 months), and IOP data at 1, 3, and 6 months postoperatively were largely missing because of incomplete clinical records. Therefore, we were unable to comprehensively assess the dynamic changes in IOP during the treatment period.

In summary, OCTA and mfERG showed that the intravitreal injection of conbercept achieved therapeutic effects similar to those of ranibizumab for the treatment of ICNV but with fewer injections, which reduced the treatment burden on patients. The use of a 1+PRN regimen for both drugs, rather than a 3-month loading dose for ranibizumab, might have affected the comparative efficacy. It is possible that a 3-month loading dose of ranibizumab could have led to better outcomes or required even fewer subsequent injections. Therefore, our conclusion that conbercept requires fewer injections should be interpreted with caution in the context of this specific dosing strategy. Our mfERG analysis was limited to P1 amplitude density because N1 implicit time was not reliably captured for all patients due to the retrospective nature of the study and inconsistent storage of raw trace data. Consequently, we could not assess changes in response speed, which might have provided additional insight into functional recovery. Future prospective studies should include both amplitude and implicit time parameters for a more comprehensive functional evaluation. The choice of conbercept or ranibizumab in this study was not randomized but was based on a combination of the physician’s preference and the patient’s economic considerations. This non-randomized assignment may have introduced selection bias. For example, patients with better economic status might have been more likely to receive a particular drug, and physicians might have preferred one drug for patients with more severe disease. Such confounding factors could affect the comparability between the two groups and limit the generalizability of our findings. Future prospective randomized controlled trials or studies using propensity score matching are needed to confirm our results.

## Data Availability

The datasets used or analyzed during the current study are available from the corresponding author on reasonable request.

## References

[B1] Bing L. Xiongze Z. Yuting P. Lan Mi. Feng W. (2018). Etiologies and characteristics of choroidal neovascularization in young Chinese patients. Ophthalmologica 241 (2), 73–80. 10.1159/000492133 30153680

[B2] Chen C. Yan M. Huang Z. Song Y. P. (2020). The evaluation of a two-year outcome of intravitreal conbercept versus ranibizumab for pathological myopic choroidal neovascularization. Curr. Eye Res. 45, 1415–1421. 10.1080/02713683.2020.1742357 32191134

[B3] Chu Y. Chen N. Yu H. Mu H. He B. Hua H. (2017). Topical ocular delivery to laser-induced choroidal neovascularization by dual internalizing RGD and TAT peptide-modified nanoparticles. Int. J. Nanomedicine 12, 1353–1368. 10.2147/IJN.S126865 28260884 PMC5325139

[B4] Cohen S. Y. Laroche A. Leguen Y. Soubrane G. Coscas G. J. (1996). Etiology of choroidal neovascularization in young patients. Ophthalmology 103, 1241–1244. 10.1016/s0161-6420(96)30515-0 8764794

[B5] David G. Lawrence J. (2006). Vision loss in younger patients: a review of choroidal neovascularization. Optom. Vis. Sci., 83(5), 316–325. 10.1097/01.opx.0000216019.88256.eb 16699445

[B6] European Medicines Agency (2016). Summary of Product character-istics. Lucentis 10 mg/mL Solution for Injection. London, United Kingdom: Novartis. Available online at: http://ec.europa.eu/health/documents/community-register/2016/20161114136324/anx_136324_en.pdf December 13, 2016).

[B7] Ho A. C. Yannuzzi L. A. Pisicano K. De Rosa J. (1995). The natural history of idiopathic subfoveal choroidal neovascularization. Ophthalmology 102, 782–789. 10.1016/s0161-6420(95)30968-2 7539905

[B8] Hoffmann M. B. Bach M. Kondo M. Li S. Walker S. Holopigian K. (2021). ISCEV standard for clinical multifocal electroretinography (MfERG) (2021 update). Doc. Ophthalmol. 142, 5–16. 10.1007/s10633-020-09812-w 33492495 PMC7906932

[B9] Huang J. Li Y. Chen Y. You Y. Niu T. Zou W. (2021). Multifocal electroretinogram can detect the abnormal retinal change in early stage of Type2 DM patients without apparent diabetic retinopathy. J. Diabetes Res. 2021, 6644691. 10.1155/2021/6644691 33681384 PMC7925060

[B10] Inoue M. Kadonosono K. Watanabe Y. Sato S. Kobayashi S. Yamane S. (2010). Results of 1-year follow-up examinations after intravitreal bevacizumab administration for idiopathic choroidal neovascularization. Retina 30, 733–738. 10.1097/IAE.0b013e3181c9699c 20168271

[B11] Jia Y. Bailey S. T. Wilson D. J. Tan O. Klein M. L. Flaxel C. J. (2014). Quantitative optical coherence tomography angiography of choroidal neovascularization in age-related macular degeneration. Ophthalmology 121, 1435–1444. 10.1016/j.ophtha.2014.01.034 24679442 PMC4082740

[B12] Kumar A. Prakash G. Singh R. P. (2004). Transpupillary thermotherapy for idiopathic subfoveal choroidal neovascularization. Acta Ophthalmol. Scand. 82, 205–208. 10.1046/j.1600-0420.2004.00217.x 15043542

[B13] Kun L. Yanping S. Gezhi X. Ye J. Wu Z. Liu X. (2019). Conbercept for treatment of neovascular age-related macular degeneration: results of the randomized phase 3 PHOENIX study. Am. J. Ophthalmol. 197, 156–167. 10.1016/j.ajo.2018.08.026 30148987

[B14] Lai T. Y. Y. Staurenghi G. Lanzetta P. Holz F. G. Melissa Liew S. H. Desset-Brethes S. (2018). Efficacy and safety of ranibizumab for the treatment of choroidal neovascularization due to uncommon cause: twelve-month results of the minerva study. Retina 38, 1464–1477. 10.1097/IAE.0000000000001744 28704254 PMC6086222

[B15] Li F. Sun M. Guo J. Ma A. Zhao B. (2017). Comparison of conbercept with ranibizumab for the treatment of macular edema secondary to branch retinal vein occlusion. Curr. Eye Res. 42, 1174–1178. 10.1080/02713683.2017.1285943 28441077

[B16] Li J. Wang W. Zhang X. Liu J. Zhang H. Cui T. (2022). Morphological and functional features in patients with idiopathic macular hole treatment. Int. J. Gen. Med. 15, 4505–4511. 10.2147/IJGM.S365886 35509600 PMC9059987

[B17] Liu B. Bao L. Zhang J. (2016). Optical coherence tomography angiography of pathological myopia sourced and idiopathic choroidal neovascularization with follow-up. Med. (Baltim) 95, 95:e3264. 10.1097/MD.0000000000003264 27057880 PMC4998796

[B18] Lu X. Sun X. (2015). Profile of conbercept in the treatment of neovascular age-related macular degeneration. Drug Des. Dev. Ther. 9, 2311–2320. 10.2147/DDDT.S67536 25960634 PMC4410828

[B19] Lu H. Yue T. Liu N. Wang Z. F. Zhai G. X. Mi D. M. (2021). Efficacy of conbercept in the treatment of choroidal neovascularization secondary to pathologic myopia. Front. Med. (Lausanne) 8, 720804. 10.3389/fmed.2021.720804 34746171 PMC8566718

[B20] Nagy D. Schönfisch B. Zrenner E. Jägle H. (2008). Long-term follow-up of retinitis pigmentosa patients with multifocal electroretinography. Invest Ophthalmol. Vis. Sci. 49, 4664–4671. 10.1167/iovs.07-1360 18566474

[B21] Paris T. Stavrenia K. Penelope B. de P. Marianna T. Olympia G. (2024). Effects of dexamethasone intravitreal implant on multifocal electroretinography in diabetic macular oedema. Drug Des. Devel Ther. 18 (0), 5367–5375. 10.2147/DDDT.S477677 39624769 PMC11609416

[B22] Qi H. J. Li X. X. Tao Y. (2010). Outcome of intravitreal bevacizumab for idiopathic choroidal neovascularization in the Chinese population. Can. J. Ophthalmol. 45, 381–385. 10.1139/i10-019 20648084

[B23] Shi X. Wei W. Zhang C. (2014). Intravitreal ranibizumab therapy versus photodynamic therapy for idiopathic choroidal neovascularization: a comparative study on visual acuity, retinal and choroidal thickness. Chin. Med. J. (Engl) 127, 2279–2285. 10.3760/cma.j.issn.0366-6999.20140544 24931242

[B24] Sudhalkar A. Yogi R. Chhablani J. (2015). Anti-vascular endothelial growth factor therapy for naive idiopathic choroidal neovascularization: a comparative study. Retina 35, 1368–1374. 10.1097/IAE.0000000000000491 25830696

[B25] Wu Q. Chen X. Feng K. Liu Y. Zhang C. Zhao L. (2020a). Evaluation of efficacy and recurrence for anti-vascular endothelial growth factor therapy in idiopathic choroidal neovascularization. BMC Ophthalmol. 20, 115. 10.1186/s12886-020-01390-4 32192468 PMC7082985

[B26] Wu P. Shi D. Chen X. Feng C. Xu H. Lin P. (2020b). Long-term efficacy of intravitreal conbercept injection in the treatment of idiopathic choroidal neovascularization. J. Ocul. Pharmacol. Ther. 36, 116–121. 10.1089/jop.2019.0075 31750756

[B27] Yang F. Dou H. L. Ma Z. Li Y. L. Lu X. R. Wang X. (2010). Serum inflammatory factors in patients with idiopathic choroidal neovascularization. Ocul. Immunol. Inflamm. 18:390–394. 10.3109/09273948.2010.483315 20666680

[B28] Yin H. Fang X. Ma J. Chen M. Yang Y. Guo S. (2016). Idiopathic choroidal neovascularization: intraocular inflammatory cytokines and the effect of intravitreal ranibizumab treatment. Sci. Rep. 6, 31880. 10.1038/srep31880 27558944 PMC4997256

[B29] Zhang M. Zhang J. Yan M. Luo D. Zhu W. Kaiser P. K. (2011). A phase 1 study of KH902, a vascular endothelial growth factor receptor decoy, for exudative age-related macular degeneration. Ophthalmology 118, 672–678. 10.1016/j.ophtha.2010.08.008 21146224

[B30] Zhang H. Liu Z. L. Sun P. Gu F. (2012). Intravitreal bevacizumab for treatment of subfoveal idiopathic choroidal neovascularization: results of a 1-year prospective trial. Am. J. Ophthalmol. 153, 300–306.e1. 10.1016/j.ajo.2011.07.019 21982109

[B31] Zübeyir Y. Mustafa D. Mehmet Cem S. Hamidu H. Serpil Y. A. (2021). Impacts of intravitreal anti-VEGF therapy on retinal anatomy and neurophysiology in diabetic macular edema. Int. Ophthalmol. 41 (5), 1783–1798. 10.1007/s10792-021-01737-w 33606153

